# Biomass Allocation and Allometric Relationships Among Major Plant Formations in the Alpine Peat Swamp Wetlands of the Yellow River on the Gannon Plateau, Gansu Province, China

**DOI:** 10.3390/plants15132089

**Published:** 2026-07-05

**Authors:** Man-Ping Kang, Cheng-Zhang Zhao

**Affiliations:** 1 Qinghai Provincial Biotechnology and Analytical Test Key Laboratory, Qinghai Provincial Key Laboratory of High-Value Utilization of Characteristic Economic Plants, College of Ecological Environment and Resources, Qinghai Minzu University, Xining 810007, China; k2429024950@163.com; 2 College of Geography and Environmental Science, Northwest Normal University, Research Center of Wetland Resources Protection and Industrial Development Engineering of Gansu Province, Lanzhou 730070, China

**Keywords:** biomass allocation patterns, allometric growth relationships, peat swamp wetlands

## Abstract

Biomass allocation patterns affect plant functions across all levels, ranging from plant growth and reproduction to the quality and energy flow of entire communities. Revealing the biomass allocation and allometric growth relationships among the dominant plant formations in alpine peat swamp wetlands not only can help elucidate the life history strategies of swamp plants, but also plays a crucial role in understanding the uncertainty of plant carbon sinks in peat swamp wetlands. Based on community surveys, this study employed analysis of variance (ANOVA) and standardized major axis estimation (SMA) to analyze the species composition, biomass allocation of different organs, and allometric growth relationships of the dominant plant formation in the alpine peat swamp wetlands of the Yellow River on the Gannon Plateau, Gansu Province, China. The results showed the following: (1) Peat swamp plants can be classified into six formations dominated by *Carex muliensis*, *Blysmus sinocompressus*, *Carex atrofusca*, *Kobresia tibetica*, *Kobresia kansuensis*, and *Carex kansuensis*. Environmental filtering was identified as the primary factor influencing the distribution of formations in this region. (2) The biomass allocation ratios of the dominant plant formations were ordered as follows: root mass ratio > leaf mass ratio > stem mass ratio. There were also significant differences in the biomass allocation of roots, stems, and leaves among different plant formations. (3) Isometric growth was observed between the leaf and stem biomass of the dominant plant formations (*p* > 0.05), while allometric growth relationships existed between root/leaf biomass and root/stem biomass (*p* < 0.05), with the growth rate of root biomass (RB) being higher than that of leaf biomass (LB) and stem biomass (SB). The biomass allocation patterns and allometric growth relationships among the roots, stems, and leaves of the dominant plant formations in peat swamp wetlands reflect the environmental plasticity mechanism of functional plant traits in heterogeneous habitats. Moreover, combining optimal allocation theory and allometric growth theory can better explain the biomass variation and adaptation mechanisms of dominant plant formations in peat swamp wetlands, providing a theoretical basis for understanding the habitat adaptation patterns of plants in alpine peat swamp wetlands.

## 1. Introduction

Peat bogs develop in depressional areas with low drainage in the cold and wet environment of alpine regions [1]. Over long-term evolution, plants in peat bogs formed a suitable spatial distribution pattern and biomass distribution model for this habitat, which became an important internal stress in the formation of carbon pools in peat bogs [2,3]. To resist the environmental stress of extreme cold, plants can improve their ability to obtain restricted resources and maintain their own growth and development by adjusting their morphological characteristics and biomass allocation patterns [4]. This strategy is an important demonstration of how plants ecologically adapt to environmental heterogeneity stress [5]. Biomass—the total amount of organic matter accumulated by an individual or community over a period of time—is an important indicator of the energy acquired by an ecosystem [6], and the distribution of biomass among different organs is a central focus in plant ecology [7,8]. Leaves are important plant structures that absorb light for photosynthesis and fix carbon, while stems provide mechanical support, as well as an important channel for plants to transport water and nutrients, and roots are the components through which plants absorb water and nutrients [9,10]. The size (or proportion) of the roots, stems, and leaves relative to biomass is called biomass allocation [11]. The biomass distribution characteristics of plant roots, stems and leaves are important traits that determine the survival ability of plants under environmental changes and represent the growth and metabolism patterns and ecological strategies of plants [12]. It can reflect the investment of photosynthetic products in different organs of the plant (between different organs above ground, between above-ground organs and underground organs) to adapt to environmental conditions [13]. Scholars have compared the advantages and disadvantages of the optimal allocation hypothesis and allometric growth theory, and the combined application of the two methods can better reveal the response of plants to environmental changes and further clarify their ecological adaptation strategies [14,15,16]. It is of great significance to understand the biomass allocation and growth relationship among plant roots, stems, and leaves for further study of the mechanism of plant adaptation to the environment and to accurately estimate the productivity of plant communities and even ecosystems.

Peat swamp wetlands have environmental characteristics of cold, high humidity, long-term water accumulation, and poor local water mobility. These are the main driving factors determining the complexity of plant community structure and the heterogeneity of habitat [17,18]. To maintain the necessary physiological activities and achieve normal growth, plants improve their environmental adaptability by balancing the biomass allocated to leaves, stems, and roots to maximize limited resources such as light, nutrition, and water, and optimize their resource allocation [19,20]. The hydrological process and soil environmental conditions of a wetland are the main limiting factors for plant growth, development, and reproduction [21]. Environmental factors have important effects on the internal living conditions and habitat selection of vegetation in marsh wetland ecosystems. At present, research on wetland plant biomass mainly focuses on the distribution model of individual dominant plant biomass under different habitat conditions, especially under drought stress [22], flooded habitat [23], soil heterogeneity, and the combination of species composition [24]. From the perspective of community, there are few studies on the biomass distribution and allometric growth relationship of major plant groups in alpine peat swamp. This study aims to accurately and quantitatively study the biomass distribution patterns and allometry relationships among roots, stems, and leaves in peat bogs. This will reveal the survival strategies and adaptive mechanisms of swamp plants and provide a theoretical basis for understanding the distribution model of carbon assimilation products and ecological adaptation strategies of plants in alpine peat swamps.

The first meander of Yellow River National Nature Reserve is an outstanding example of a primitive and representative alpine peat swamp wetland located in the Gannan Plateau. Its plants have developed unique regional ecological characteristics to adapt to the cold, waterlogged, and oxygen-poor environment, exhibiting typicality and specificity, as reported in previous studies [25,26]. Peat bog wetlands in nature reserves provide excellent opportunities for studying peat bog plant biomass characteristics, carbon storage, and environmental response mechanisms, as demonstrated in previous studies [27]. Current research on peat bogs in the first meander of Yellow River National Nature Reserve, both domestically and internationally, primarily focuses on wetland changes [28], wetland landscape evolution [29], plant community structure, and species diversity [30]. However, there are few studies on the biomass distribution and allometric growth relationship of major plant groups in alpine peat swamps. In view of this, using the plants of the peat swamp in the first meander of Yellow River National Nature Reserve as the research object, the DCA ranking method was used to classify the main plant groups of the peat swamp wetland. Analysis of variance and standardized principal axis estimation (SMA) were used to examine the biomass distribution and allometric growth relationships among major plant groups in peat bogs. In this study, we attempt to elucidate the following: (1) What is the species composition of the main flora in alpine peat swamp wetlands? (2) What are the biomass allocation strategies of the main plant groups in alpine peat bogs? (3) What characterizes and determines the allometric growth relationship of root, stem, and leaf organs of major plant groups in alpine peat swamp wetland? This study aims to reveal the main resource allocation models and survival strategies of peat marsh wetland from the perspective of community science, and it deepens the understanding of plant biomass allocation strategies and resource niches in peat marsh wetland. It provides a theoretical basis for the investigation of the adaptation mechanism of plant and environment synergistic change in peat bog wetland.

## 2. Methods

### 2.1. Study Sites and Sampling

The first meander of Yellow River National Nature Reserve in the study area is located in the southwestern part of Maqu, Gannan Tibetan Autonomous Prefecture, Gansu Province. The study area experiences long and frigid winters, short and mild summers, and exhibits a small annual temperature range and large daily temperature variations. The mean annual temperature of the area ranges from 1.1 to 1.5 °C. The study area receives an average annual rainfall of 635–655 mm, while the evaporation rate averages at 1353.4 mm per year. The annual sunshine duration in the study area average is approximately 2580 h. The study area has an average frost-free period of 19 days and experiences 190 consecutive days of soil freezing. The soil types in the study area are primarily meadow soils, swamp soils, and peat soils. The dominant plant species found in the bog vegetation are herbaceous sedges such as *Blysmus sinocompressus*, *Ranun culus hirtellus*, *Kobresia tibetica*, *Carex muliensis*, *Potentilla anserina*, *Carex kansuensis*, *Sanguisorba filiformis*, *Koeleria cristata*, *Heleocharis dulcis*, *Pedicularis kansuensis*, and *Polygonum viviparum*. The plant community in the study area is diverse and species-rich, with relatively protected and intact natural vegetation. It is typical and representative of the high-altitude regions in China, and is an ideal area for studying the biomass allocation strategies and environmental adaptation strategies of peat swamp plant communities.

### 2.2. Experimental Setup

**Figure 1:**
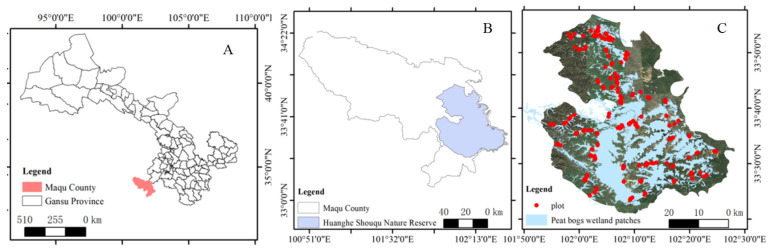
The location and setting of experimental plots in the first meander of Yellow River National Nature Reserve of Maqu County: (**A**) the location of Maqu in Gansu Province; (**B**) the position of the Huanghe Shouqu Nature Reserve in Maqu; (**C**) the 189 sampling points set up along the Huanghe Shouqu.

The distribution of peat bogs was identified based on Google Maps (https://www.google.com/maps/@22.3527242,114.1394,11z?entry=ttu&g_ep=EgoyMDI2MDYyOS4wIKXMDSoASAFQAw%3D%3D, accessed on 1 July 2026) and high-resolution One Satellite Remote Sensing Image data. The area with relatively intact peat bog preservation and less human activity interference in the Huanghe First Curve International Important Wetland was selected as the research object. Considering multiple on-site investigations from 2021 to 2022 in July and August, and taking into account accessibility and all wetland types, plant types, landform types, altitude, slope, and aspect of the terrain conditions in the study area, 189 survey plots were set up for the peat bog patches in this area. From July to August 2023, the 189 survey plots were investigated and sampled for plant communities and soil physical and chemical properties (Figure 1). During the experiment, the plant growth was good, and the plant community and wetland plant observation data were comparable. The primary focus of the field investigation included a comprehensive assessment of key flora, plant species diversity, wetland classifications, geomorphic features, water supply sources for peat bogs, soil types, and other relevant information. Handheld GPS devices were utilized to accurately record survey point locations; detailed descriptions and photographs were also collected according to the established investigation criteria. The study area predominantly consists of winter pasture where plant growth was robust during the experimental period. This favorable condition helped mitigate grazing interference factors, ensuring that the experimental data obtained are comparable across different observations.

### 2.3. Survey Sampling

(1) Community survey: Based on the “Flora of China” and the “Plant Catalogue”, confirm and name all the species present in each 1 m × 1 m sample plot, and record and organize the information; measure and record the height, coverage, and density of all plants in the sample plot. Then, cut the above-ground part of the 30 cm (length) × 30 cm (width) plants to the ground, and determine the wet weight on the spot and put the parts into a labeled, site-specific envelope that is numbered and marked with the vegetation type.
(2) Litter collection: After the community survey and the collection of above-ground samples are completed, collect the amount of litter in the survey sample plot (30 m × 30 m), remove the surface adhering soil, sand, and other impurities, and put it into envelopes and bring it back to the laboratory.
(3) Underground biomass collection: Use the trench method to dig underground biomass samples within the sample plot at a depth of 30 cm (length) × 30 cm (width) × 50 cm (height). During the initial collection, the root depth of the plant was determined by sampling to a depth of 100 cm (the root system of the wetland community was mostly distributed in the 0–50 cm soil layer). The collected underground plant tissues were placed in a nylon mesh bag with an aperture of 0.25 mm and washed with water to clean the attached soil, and then the wet weight was weighed. Following this, the entire plant root system was packed into envelopes, numbered, and labeled with the type of vegetation, and the above- and below-ground parts of the plant were brought back to the laboratory.

### 2.4. Analysis of Laboratory Experiment Data

In the Laboratory of Ecology and Hydrology of Northwest Normal University, the above-ground plant elements (divided into stems and leaves) and the underground portions (roots) of the plant community collected in the field were respectively put into numbered envelopes. First, the sample was heated at 105 °C for 30 min, then dried in an oven at 85 °C for 24 h until a constant weight was achieved. The weight was measured using an electronic balance (with an accuracy of 0.0001 g), and the biomass of the plant community’s stems, leaves, and roots were recorded. The biomass allocation parameters of individual roots, stems, and leaves of plants were based on the proportion of the mass of individual roots, stems, and leaves to the total mass of the plant [7].

### 2.5. Data Analysis

Importance value is a comprehensive quantitative measure of the importance of a species in its community. Significance values for each species are calculated using the average of relative height, relative coverage, relative density, and relative biomass. As a comprehensive quantitative index to evaluate the relative importance of each species in a community, the importance value (IV) is calculated as follows:

Relative height = (Average height of a species in the quadrat/Sum of average heights of all species in the quadrat) × 100%

Relative coverage = (Coverage of a species in the quadrat/Sum of coverage of all species in the quadrat) × 100%

Relative density = (Density of a species in the quadrat/Sum of density of all species in the quadrat) × 100%

Relative biomass = (Biomass of a species in the quadrat/Sum of biomass all species in the quadrat) × 100%

$$IV=\frac{Relative\;Height+Relative\;Coverage+Relative\;density+Relative\;biomass}{4}$$

Standardized spindle estimation method

Before analyzing the biomass data of plant parts, a logarithmic transformation was performed to make it conform to a normal distribution. The biomass of roots, stems, and leaves was fitted using the allometry equation Y = β·X^α^, and logarithms were taken on both sides of the equation to convert it into a linear equation lgY = lg*β* + *α*lgX, where Y and X represent the dependent and independent variables, respectively, lg*β* is the intercept, and α is the slope. When α = 1, it means that the dependent variable and the independent variable are growing at an equal rate. If α ≠ 1, it is an allometric growth relationship. The standardized spindle estimation method (SMA) was used for analysis. The Pitman method was used to calculate the confidence interval of the slope, and the Warton and Weber (2002) [31] method was used to determine the heterogeneity of the regression slope; the common slope was calculated when the slope was homogeneous. The differences in intercept and slope were tested using ANOVA, and the parameters of the allometric growth equation were tested with software. (S)MATR Version 2.0 was used to calculate (Falster et al., 2006) [32], and the smart package of R software 4.6.1 (R Development Core Team, 2011) was used to test the significance of the difference between slope and 1.0. The significance level of all statistical tests was α = 0.05.

## 3. Analysis of the Results

### 3.1. Main Plant Formations and Species Composition of Peat Bog Wetland

#### 3.1.1. Primary Plant Formations in Peat Bog Wetland

The 189 herbaceous plots were classified using the Detrended Correspondence Analysis (DCA) method. The distribution of plant communities in the DCA sorting diagram is more concentrated; there are clear boundaries between different vegetation communities, which can reveal the basic distribution pattern of plant communities in the Yellow River Head Curve Peat Swamp Wetland and their relationship with the environment. The DCA of the peat swamp plant community at the headwaters of the Yellow River shows that the eigenvalue of axis 1 is 0.76, and the eigenvalue of axis 2 is 0.43. Based on the characteristic information of the first two sorting axes, it can be concluded that axis 1 can reflect the composition changes in the plant community under the environmental gradient (Table 1).

Combining the environmental gradient and plant quadrats, based on the DCA, the peat swamp plants were classified into six plant communities (Figure 1). The dominant species and associated species of each community were determined based on the species composition and importance values in the survey quadrats.

**Figure 2:**
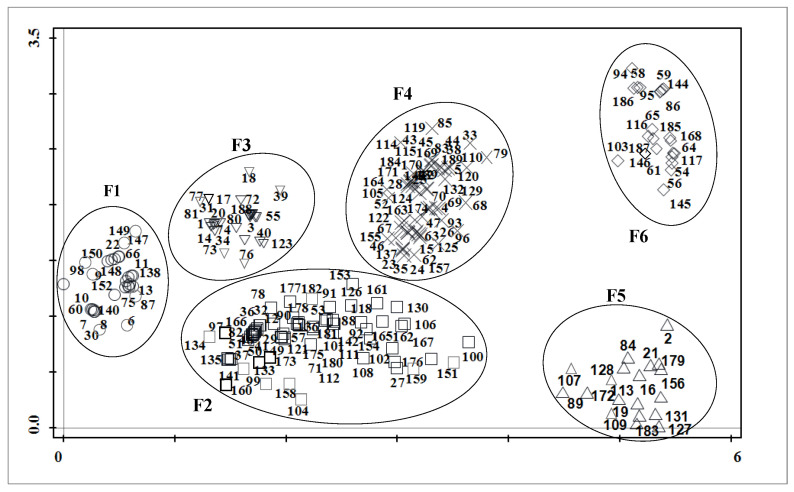
DCA sequence map of 189 plant quadrats in alpine peat bogs. Note: DCA sequence map of 189 plant quadrats in alpine peat bogs in which F1 = *Carex muliensis* community; F2 = *Blysmus sinocompressus* community; F3 = *Carex atrofusca* community; F4 = *Kobresia tibetica* community; F5 = *Kobresia kansuensis* community; and F6 = *Carex kansuensis* community.

The species composition and importance values of the alpine peat swamp plant community are presented in Table 2 and Figure 2. The F1 community is characterized by *Carex muliensis* as its dominant species and includes *Kobresia tibetica*, *Kobresia humilis*, and *Heleocharis dulcis,* along with other hydrophytic plants as its associated species. The F1 community is principally distributed in the alluvial depressions at the foot of the mountains and the intermountain depressions. The plant community is distributed in the form of patches, ridge networks, and arc-shaped grass mounds. There is seasonal or perennial water accumulation between the grass hills, with the water depth being approximately 10–15 cm (Table 2). The F2 community is primarily characterized by the plant species *Blysmus sinocompressus*. The associated species include *Kobresia tibetica*, *Carex pseuduncinoides, Ranunculus natans*, and *Carex muliensis*. This community is primarily distributed in floodplains, alluvial depressions, and the edges of marshy wetlands. The surface of the area experiences seasonal or intermittent water accumulation, with the water depth being approximately 0–10 cm. The F3 community is dominated by *Carex atrofusca*, with associated species such as *Blysmus sinocompressus*, *Scirpus pumilus*, and *Potentilla anserina*. This community is distributed in floodplains and gently sloping hillslopes in the piedmont area, forming a herbaceous marsh with intermittent water accumulation and a water depth ranging from 0 to 5 cm. F4 is characterized by *Kobresia tibetica* as its dominant species, with associated species including *Blysmus sinocompressus*, *Sanguisorba filiformis*, and *Halerpestes tricuspis*. It is distributed in fan-edge depressions, piedmont sloping lands, and intermontane depressions at an altitude of 3550–3750 m, forming marshy meadows with intermittent waterlogging and a water table depth of approximately −10 to 5 cm. The F5 community has *Kobresia kansuensis* as the dominant species, accompanied by associated species including *Kobresia tibetica*, *Ligularia virgaurea*, *Poa pratensis*, and *Scirpus pumilus*. It is distributed in the lower river terraces and the foot of shady slopes at an altitude of 3400–3500 m. The surface is moist without waterlogging, with a water table depth of about −15 to 0 cm. The F6 community is dominated by *Carex kansuensis* as the dominant species, with associated plants such as *Kobresia tibetica*, *Carex muliensis*, *Pedicularis kansuensis,* and *Cremanthodium brunneopilosum*. It is mainly distributed in piedmont sloping lands at an altitude of 3650–3800 m. The surface is moist without waterlogging, with a water table depth of approximately −15 to 0 cm.

#### 3.1.2. Species Composition and Importance Value Characteristics of Main Vegetation Formations

**Figure 3:**
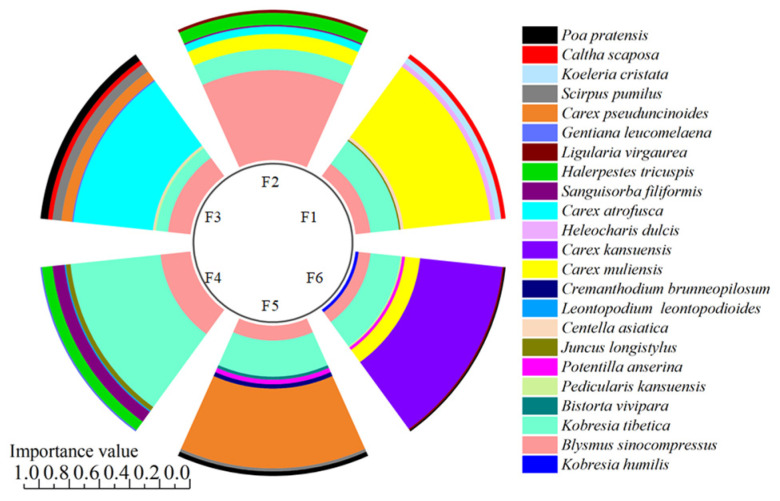
Importance values of species in main plant formations of peat bog wetlands. Note: In alpine peat bogs in which F1 = *Carex muliensis* community; F2 = *Blysmus sinocompressus* community; F3 = *Carex atrofusca* community; F4 = *Kobresia tibetica* community; F5 = *Kobresia kansuensis* community; and F6 = *Carex kansuensis* community.

Based on the calculation of plant importance values (IV), the species composition of the main plant formations in the peat bog wetlands is as follows (Figure 3): In F1, *Carex muliensis* has the highest importance value (0.51), followed by associated species such as *Kobresia tibetica* (0.38), *Kobresia humilis* (0.30), *Equisetum fluviatile* (0.32), *Blysmus sinocompressus*, *Eleocharis valleculosa* var. setosa (0.22), and *Centella asiatica* (0.17). In F2, *Blysmus sinocompressus* has the largest importance value (0.63), with associated species including *Kobresia tibetica* (0.47), *Carex muliensis* (0.36), *Halerpestes tricuspis* (0.24), *Centella asiatica* (0.17), *Carex atrofusca* (0.13), and *Juncus przewalskii* (0.10). In F3, *Carex atrofusca* has the maximum importance value (0.58), accompanied by associated species such as *Blysmus sinocompressus* (0.42), *Kobresia kansuensis* (0.26), *Potentilla anserina* (0.20), *Kobresia tibetica* (0.19), *Caltha scaposa* (0.16), *Poa annua* (0.13), and *Polygonum viviparum* (0.10). In F4, *Kobresia tibetica* has the highest importance value (0.60), followed by associated species such as *Blysmus sinocompressus* (0.50), *Sanguisorba filiformis* (0.30), *Halerpestes tricuspis* (0.28), *Potentilla anserina* (0.15), and *Scirpus pumilus* (0.12). In F5, *Kobresia kansuensis* has the highest importance value (0.63), with associated species including *Kobresia tibetica* (0.48), *Polygonum viviparum* (0.10), *Scirpus pumilus* (0.26), *Poa pratensis* (0.24), *Potentilla anserina* (0.16), and *Carex kansuensis* (0.10). In F6, *Carex kansuensis* has the largest importance value (0.53), accompanied by associated species such as *Kobresia tibetica* (0.47), *Carex muliensis* (0.31), *Pedicularis kansuensis* (0.21), *Ligularia virgaurea* (0.16), and *Blysmus sinocompressus* (0.20).

### 3.2. Biomass Allocation Characteristics of Various Organs in Main Plant Formations

**Figure 4:**
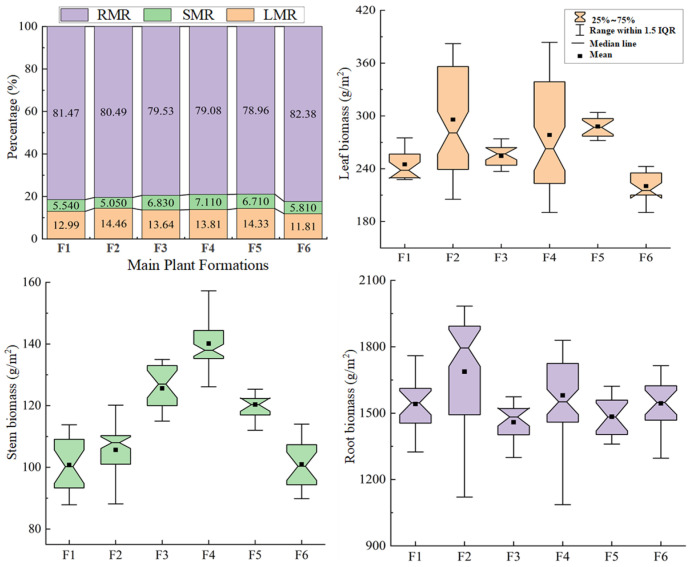
Biomass allocation of main plant formations in alpine peat bog wetlands. Note: F1 = *Carex muliensis* formation; F2 = *Blysmus sinocompressus* formation; F3 = *Carex atrofusca* formation; F4 = *Kobresia tibetica* formation; F5 = *Kobresia kansuensis* form; F6 = *Carex kansuensis* community. RMR = root mass ratio; LMR = leaf mass ratio; SMR = stem mass ratio.

Differences in biomass allocation among the root/stem/leaf organs of main plant formations in alpine peat bog wetlands are shown in Figure 4. The order of plant biomass allocation ratio was root mass ratio (RMR) > leaf mass ratio (LMR) > stem mass ratio (SMR). Among the peat bog plants, the root mass ratio was the highest. In particular, the *Carex kansuensis* formation (F6) and *Carex muliensis* formation (F1) had the largest root mass ratios, with values of 82.38% and 81.47%, respectively, while the *Kobresia kansuensis* formation (F5) had the smallest root mass ratio (78.96%). For the leaf mass ratio of peat bog plant formations, the *Blysmus sinocompressus* formation (F2) had the highest value (14.46%), and the *Carex kansuensis* formation (F6) had the smallest (11.81%). The stem mass ratio of plant formations in peat bog wetlands was the smallest, ranging from 5.34% to 7.0%. The *Kobresia tibetica* formation (F4) had the highest stem mass ratio (7.11%), followed by the *Kobresia kansuensis* formation (F5) and *Carex atrofusca* formation (F3) with stem mass ratios of 6.71% and 6.83%, respectively. The *Blysmus sinocompressus* formation (F2) had the smallest stem mass ratio (5.05%).

There were differences in organ tissue biomass among the main plant formations in peat bogs (Figure 4). Among them, the *Blysmus sinocompressus* formation (F2) had the highest root biomass and leaf biomass, with values of 1687.56 ± 45.67 g/m^2^ and 295.87 ± 20.45 g/m^2^, respectively. The *Carex atrofusca* formation (F3) had relatively low root biomass (1459.10 ± 61.87 g/m^2^). The above-ground parts (stems and leaves) of the *Carex kansuensis* formation (F6) were relatively low, with values of 100.76 ± 17.37 g/m^2^ (stem biomass) and 220.33 ± 15.63 g/m^2^ (leaf biomass). The *Kobresia tibetica* formation (F4) had relatively high stem biomass (140.15 ± 18.43 g/m^2^).

### 3.3. Allometric Growth Relationships of Organ Biomass in Plant Formations

#### 3.3.1. Allometric Growth Relationship Between Leaf Biomass and Stem Biomass

**Figure 5:**
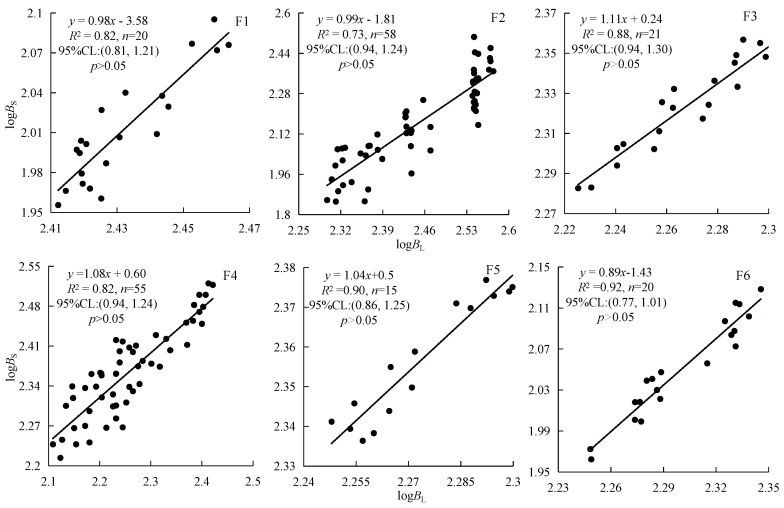
Allometric growth relationship between leaf and stem biomass of main plant formations in alpine peat bogs. Note: F1 = *Carex muliensis* formation; F2 = *Blysmus sinocompressus* formation; F3 = *Carex atrofusca* formation; F4 = *Kobresia tibetica* formation; F5 = *Kobresia kansuensis* formation; F6 = *Carex kansuensis* formation. LogBL = leaf biomass; LogBS = stem biomass.

As shown in Figure 5, there was a positive correlation between the leaf biomass and stem biomass of the main plant formations in alpine peat bog wetlands (*p* < 0.05). Through standard major axis (SMA) analysis, significant differences were observed in the slopes between leaf biomass and stem biomass among the main plant formations (*p* < 0.05). For the *Carex muliensis* formation (F1), *Blysmus sinocompressus* formation (F2), and *Carex kansuensis* formation (F6), the SMA slopes were 0.98, 0.99, and 0.89, respectively, with no significant differences detected when compared with 1.0 (*p* > 0.05). Their 95% confidence intervals (CI) were (0.81, 1.21), (0.94, 1.24), and (0.77, 1.01), respectively. These results indicate that leaf biomass and stem biomass in F1, F2, and F6 followed an isometric growth relationship, with the growth rate of leaf biomass being significantly higher than that of stem biomass.

For the *Carex atrofusca* (F3), *Kobresia tibetica* (F4), and *Kobresia kansuensis* (F5) formations, the SMA slopes were 1.11, 1.08, and 1.04, respectively, with no significant differences from 1.0 (*p* > 0.05). Their 95% confidence intervals were (0.94, 1.30), (0.94, 1.24), and (0.86, 1.25), respectively. This suggests an isometric growth relationship between leaf biomass and stem biomass in F3, F4, and F5 as well. Overall, the leaf biomass and stem biomass of the main plant formations in alpine peat bogs exhibited isometric growth, reflecting an ecological strategy of balanced growth.

#### 3.3.2. Allometric Growth Relationship Between Leaf Biomass and Root Biomass

**Figure 6:**
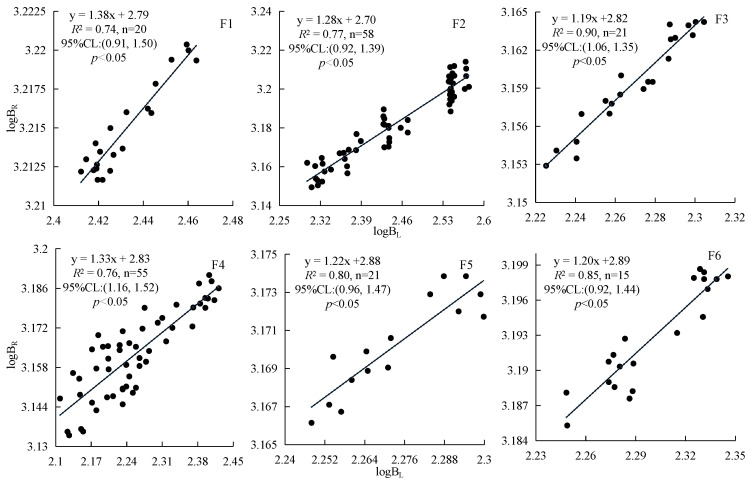
Allometric growth relationship between leaf and root biomass of main plant formations in alpine peat bogs. Note: F1 = *Carex muliensis* formation; F2 = *Blysmus sinocompressus* formation; F3 = *Carex atrofusca* formation; F4 = *Kobresia tibetica* formation; F5 = *Kobresia kansuensis* formation; F6 = *Carex kansuensis* formation. LogBL = leaf biomass; LogBR = root biomass.

**Figure 7:**
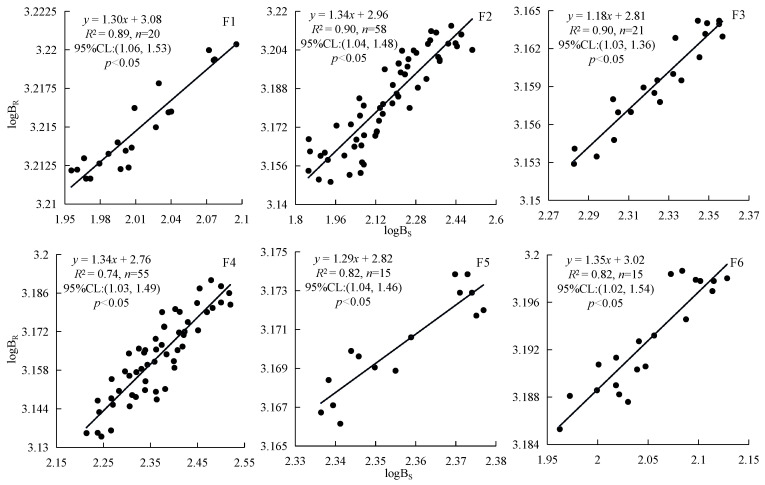
Allometric growth relationship between stem and root biomass of main plant formations in alpine peat bogs. Note: F1 = *Carex muliensis* formation; F2 = *Blysmus sinocompressus* formation; F3 = *Carex atrofusca* formation; F4 = *Kobresia tibetica* formation; F5 = Kobresia kansuensis formation; F6 = *Carex kansuensis* formation. LogBS = stem biomass; LogBR = root biomass.

As shown in Figure 6, there was a significant positive correlation between the leaf biomass and root biomass of the main plant formations in alpine peat bogs (*p* < 0.05). Through standard major axis (SMA) analysis, significant differences were found in the slopes between leaf biomass and root biomass among different plant formations (*p* < 0.05). Among them, the *Carex muliensis* formation (F1) had the highest SMA slope (1.38, 95% confidence interval: CI = (0.91, 1.50)), followed by the *Kobresia tibetica* formation (F4) with a relatively large SMA slope of 1.33 (95% confidence interval: CI = (1.16, 1.52)). The SMA slopes of the *Blysmus sinocompressus* formation (F2) and *Kobresia kansuensis* formation (F5) were 1.28 (95% confidence interval: CI = (0.92, 1.39)) and 1.22 (95% confidence interval: CI = (0.96, 1.47)), respectively. The SMA slopes of the *Carex atrofusca* formation (F3) and *Carex kansuensis* formation (F6) were 1.19 (95% confidence interval: CI = (1.06, 1.35)) and 1.20 (95% confidence interval: CI = (0.92, 1.44)), respectively. The allometric growth exponents of leaf biomass and root biomass for all plant formations were significantly greater than 1.0 (Figure 7, *p* < 0.05), indicating an allometric growth relationship. Moreover, the growth rate of root biomass in the plant formations was higher than that of leaf biomass.

#### 3.3.3. Allometric Growth Relationship Between Stem Biomass and Root Biomass

As shown in Figure 7, there was a significant positive correlation between the stem biomass and root biomass of the main plant formations in alpine peat bogs (*p* < 0.05), and significant differences existed in the slopes between stem biomass and root biomass among different plant formations (*p* < 0.05).

Among them, the *Blysmus sinocompressus* formation (F2) and *Kobresia tibetica* formation (F4) had the largest SMA slopes, with values of 1.34 (95% confidence interval: CI = (1.04, 1.48)) and 1.34 (95% confidence interval: CI = (1.03, 1.49)), respectively (note: the slope value of F4 was supplemented to maintain data consistency based on contextual logic). These were followed by the *Carex kansuensis* formation (F6) and *Carex muliensis* formation (F1), which had relatively large SMA slopes of 1.35 (95% confidence interval: CI = (1.02, 1.54)) and 1.30 (95% confidence interval: CI = (1.06, 1.53)), respectively. The SMA slope of the *Kobresia kansuensis* formation (F5) was 1.29 (95% confidence interval: CI = (1.04, 1.46)), while the *Carex atrofusca* formation (F3) had the lowest SMA slope of 1.18 (95% confidence interval: CI = (1.03, 1.36)). The allometric growth exponents of stem biomass and root biomass for all plant formations were significantly greater than 1.0 (Figure 6, *p* < 0.05), indicating an allometric growth relationship between the two. Furthermore, the growth rate of root biomass in the plant formations was higher than that of stem biomass.

## 4. Discussion

### 4.1. Biomass Characteristics of Main Plant Communities and Their Primary Plant Part Biomass Allocations

In alpine peat bog wetlands, the environment characterized by long-term waterlogging and poor local water mobility leads to soil environmental heterogeneity and complexity in the plant community structure. To maintain essential physiological activities and achieve normal growth, plants optimize their resource allocation by balancing the biomass allocated to roots, stems, and leaves, thereby enhancing their adaptability to the environment. It was observed in this study that the order of biomass allocation ratio among the main plant formations in alpine peat bogs was root mass ratio (RMR) > leaf mass ratio (LMR) > stem mass ratio (SMR). This phenomenon is mainly attributed to the following two reasons: (1) The constructive species of peat bog wetland plants are short-rhizome, dense-tussock perennial Cyperaceae species, such as *Blysmus sinocompressus*, *Kobresia tibetica*, *Carex muliensis*, and *Carex kansuensis* (Figure 2). In waterlogged environments, to obtain oxygen from the surface, these plants grow new shoots upward and adventitious roots downward through shortened underground rhizomes, allocating more biomass to clonal reproductive organs to improve nutrient use efficiency. The preferential allocation of biomass to roots supports the optimal allocation hypothesis, which is consistent with the research conclusions of Reich et al. (2014) [33]. (2) In peat bogs, the water level is close to the surface, and the soil is under anaerobic decomposition conditions due to long-term waterlogging. A large amount of undecomposed or semi-decomposed organic residues exist in the soil, which may be the reason for the high biomass of underground parts (roots). The large underground biomass of peat bog plants results in a relatively low proportion of biomass of above-ground parts (stems and leaves).

The allocation pattern of plant biomass mainly depends on plant species, individual plant development, and their living environment. This study found that there were differences in biomass allocation among different plant formations: the *Carex kansuensis* formation (F4) had the largest root mass ratio (82.78%) and a relatively low leaf mass ratio (11.81%); the *Blysmus sinocompressus* formation (F2) had the highest leaf mass ratio (14.63%) and a relatively low stem mass ratio; and the *Kobresia tibetica* formation (F4) had the highest stem mass ratio (7.0%) (Figure 4). These differences are mainly due to the following reasons: (1) The *Carex kansuensis* formation (F6) is of the rhizome/tussock type. Its roots extend laterally, with extremely strong penetration ability of root tips and root caps, as well as strong self-propagation ability, resulting in large root biomass. Moreover, this formation is distributed in piedmont sloping lands at relatively high altitudes, where soil temperature is lower and the availability of effective resources such as soil nutrients and water is reduced. To acquire environmental resources more efficiently, plants invest limited photosynthates in roots to expand the distribution range of roots for soil resource absorption, thereby enhancing the ability of roots to obtain nutrients [7]. In addition, the Gannan plateau has strong ultraviolet radiation, and low temperatures inhibit plant photosynthesis and growth—for evergreen gymnosperms especially, the leaf metabolic rate is significantly reduced. This leads to a decrease in the biomass allocated by plants to leaves [19]. Therefore, the *Carex kansuensis* formation (F6) has the largest root mass ratio (82.78%) and a relatively low leaf mass ratio (11.81%) (Figure 4). (2) The *Blysmus sinocompressus* formation (F2) is distributed at the edge of marsh wetlands at relatively low altitudes, where hydrothermal conditions are better. Plants in this formation grow in clusters with high coverage and density. Under high density, the shading degree between plants is significant, intensifying the competition for light resources. To adapt, plants allocate more nutrient resources to leaves and stems: they increase specific leaf area and plant height to optimize photosynthesis. Additionally, July to August is the peak growth period of plants, during which more biomass is invested in leaves, which reflects the plant’s growth cycle characteristics. Hence, the *Blysmus sinocompressus* formation (F2) has a higher leaf mass ratio compared to other formations. (3) The constructive species of the *Kobresia tibetica* formation (F4), *Kobresia tibetica*, is a perennial cyperaceae species with dense clumping culms. Its culms are thick, hard, and upright. When the survival rate of above-ground parts is guaranteed, the plant enhances the expansion ability of shoots to expand the range of light energy acquisition. Furthermore, plants need to balance limited resources to achieve the most favorable state for reproductive growth: this includes meeting the nutritional and water transport requirements of leaves, ensuring the safety of water transport, and maximizing the water conduction efficiency of vessels in culms. To achieve these goals, plants increase the biomass allocated to stems. Enhancing stem biomass allocation is crucial for improving the establishment efficiency of *Kobresia tibetica,* explaining why the *Kobresia tibetica* formation (F4) has the highest stem mass ratio (7.0%) among all plant formations.

### 4.2. Allometric Growth Relationships of Organ Biomass in Main Plant Formations

Plant allometric growth patterns are biological rules with certain genetic characteristics, and exhibit plastic responses to the environment. The allometric growth of plants is closely related to their biomass allocation, resource utilization, and morphological adaptation to heterogeneous environments. Among plant organs, leaves and stems are key organs for material production and conduction. Their growth patterns directly determine the plant’s resource allocation and photosynthetic efficiency, which in turn affect its ability to intercept light and acquire carbon. In this study, it was found that there was an isometric growth relationship between leaf and stem biomass in the alpine peat bog plant communities (*p* > 0.05), consistent with the findings of Cheng et al. (2014) [34], indicating that plants adopt an ecological strategy of balanced growth. Alpine peat bog wetlands are characterized by long-term waterlogging and low temperatures, forming an anaerobic environment with low microbial activity. As a result, a relatively low nutrient availability is required for plant growth. To acquire environmental resources more efficiently, wetland plants invest limited photosynthates in roots to expand the distribution range of root absorption for soil resources, thereby enhancing the ability of roots to obtain nutrients [35]. In addition, the constructive species of wetland plants are of the rhizome/tussock type: their roots extend laterally, with extremely strong penetration ability of root tips and root caps, as well as strong self-propagation ability, leading to large root biomass. Therefore, there is an allometric growth relationship between root biomass and leaf/stem biomass, with the growth rate of root biomass being higher than that of leaf and stem biomass. This suggests that these plants tend to allocate more resources to roots to improve nutrient acquisition capacity. This result is consistent with the study by Wu et al. (2013) [36], who found that biomass allocation in alpine grasslands supports the allometric allocation hypothesis rather than the isometric allocation hypothesis. Furthermore, the allometric growth relationships of alpine peat bog plants are similar to those of herbs in dry/hot valleys and alpine shrub plants. In this study, there was a convergence trend in the allocation patterns among coexisting species, while a divergence trend in allocation patterns seemed to exist among the constructive species of different plant formations. Therefore, different species may occupy different environments through niche differentiation during the evolutionary process, and species are also important factors affecting the allometric growth relationships of plant organs.

The allometric growth relationships of plant organs may be the result of adaptation of different species to specific environmental conditions. Allometric growth relationships of organs in plant species adapted to different environments will inevitably differ [37]. The six plant formations in this study belong to the genera *Kobresia* and *Carex* (Cyperaceae), and significant differences were observed in their biomass allocation ratios and allometric growth relationships. Among them, the *Carex muliensis* formation (F1), *Blysmus sinocompressus* formation (F2), and *Carex kansuensis* formation (F6) exhibited an isometric growth relationship between leaf biomass and stem biomass, with the growth rate of leaf biomass being significantly higher than that of stem biomass (Figure 5). This is mainly because the constructive species of F1, F2, and F6 are *Carex muliensis*, *Blysmus sinocompressus*, and *Carex kansuensis*, respectively, all of which are high-quality forages on the plateau. These plants grow in clusters; under high density, the elongation of individual plant leaves allows the plants to intercept more light resources and synthesize more photosynthates. The accumulation of leaf biomass can be compensated by leaf elongation, thereby achieving a relatively stable balance between the supply and demand of light resources. As a result, the rate of increase in leaf dry matter content is significantly elevated. This sensing mechanism and phenotypic plasticity are formed by plants through long-term evolution, which can improve their own fitness. Therefore, plants allocate as much nutrient resources as possible to leaves and roots for photosynthesis and nutrient absorption. At the same time, they reduce resource allocation to stems, which may be due to the relatively weak light competition they face in their habitats. The constructive species of the *Carex atrofusca* formation (F3), *Kobresia tibetica* formation (F4), and *Kobresia kansuensis* formation (F5) are of the dense clumping culm type, with thick and upright culms. These formations are distributed in areas with relatively low altitudes, where plant growth occurs in regions with higher productivity. Under high density, plants allocate more biomass to culms to intercept more light resources and synthesize more photosynthates, leading to potentially higher light competition intensity [38]. Consequently, these three plant formations show an isometric growth relationship between leaf biomass and stem biomass, with the growth rate of stem biomass being significantly higher than that of leaf biomass (Figure 6). This is consistent with the research results of Mitra (2011) [39], indicating that the allometric growth relationships between plant organs may be an adaptation of plants under natural selection. During the growth of plant communities, constructi with the findings of e species expand their territories by increasing dry matter investment in roots. The biomass relationships among roots, stems, and leaves of peat bog plants reflect the balance between photosynthetic efficiency, costs, and benefits. This not only reveals the specific ecological adaptation strategies of populations under adverse conditions but also illustrates the impact of resource reallocation caused by disturbances on the structure and function of ecosystems.

## 5. Conclusions

Alpine peat bog wetlands are rich in plant species. Based on community survey and species importance value, the plants were classified into six formations, with *Carex muliensis*, *Blysmus sinocompressus*, *Carex atrofusca*, *Kobresia tibetica*, *Kobresia kansuensis*, and *Carex kansuensis* as the constructive species for the groups. Environmental filtering is the main factor affecting the community distribution in this region. Since the water level of peat bog wetlands is close to the surface, plants grow new shoots upward and adventitious roots downward to obtain oxygen from the surface and allocate more biomass to roots to improve nutrient use efficiency. As a result, the order of plant biomass allocation ratio is root mass ratio (RMR) > leaf mass ratio (LMR) > stem mass ratio (SMR), which supports the optimal allocation hypothesis. The allometric relationships among root, stem, and leaf biomasses for peat bog plants reflect the adaptive strategies of plants to heterogeneous habitats: There is an isometric growth relationship between leaf and stem biomass of marsh plants, indicating an adoption of a balanced growth strategy. There are allometric growth relationships between root biomass and both leaf and stem biomass. In the low-temperature and waterlogged environment of peat bogs, constructive species invest more dry matter in roots to expand their territories during growth, thereby enhancing their ability to acquire water and nutrients. Consequently, in these plants, the rate of root biomass growth is higher than that of stem and leaf biomass.

## Figures and Tables

**Table 1:** DCA ranking characteristic statistics of 189 quadrats in alpine peat bogs.

	Sorting Axis			
	DCA1	DCA2	DCA3	DCA4
Eigenvalue	0.76	0.43	0.25	0.11
DCA value	20.77	32.43	39.34	42.4
Axial length	5.49	3.07	2.94	1.94

**Table 2:** Species composition and habitat environment of major plant groups in the study area peat bogs.

Flora	Depth to Groundwater Table (cm)	Altitude (m)	Dominant Species	Accompanying Species
F1	10–15	3450–3750	*Carex muliensis*	*Kobresia tibetica*, *Kobresia humilis*, *Equisetum fluviatile*, *Blysmus* *sinocompressus*, *Eleocharis valleculosa* var. setosa, *Centella* *asiatica*, and associated species.
F2	0–10	3400–3600	*Blysmus sinocompressus*	*Kobresia tibetica*, *Carex muliensis*, *Halerpestes tricuspis*, *Centella asiatica*, *Carex atrofusca*, *Juncus przewalskii*, and associated species.
F3	0–5	3550–3700	*Carex atrofusca*	*Blysmus sinocompressus*, *Kobresia kansuensis*, *Potentilla anserina*, *Kobresia tibetica*, and associated species.
F4	−10–5	3550–3750	*Kobresia tibetica*	*Blysmus sinocompressus*, *Sanguisorba filiformis*, *Halerpestes tricuspis*, *Potentilla anserina*, and associated species.
F5	−10–0	3400–3500	*Kobresia kansuensis*	*Kobresia tibetica*, *Polygonum viviparum*, *Scirpus pumilus*, *Poa pratensis*, *Potentilla anserina*, and associated species.
F6	−15–0	3650–3800	*Carex kansuensis*	*Kobresia tibetica*, *Carex muliensis*, *Pedicularis kansuensis*, and associated species.

Depth to groundwater table.

## Data Availability

The original contributions presented in this study are included in the article. Further inquiries can be directed to the corresponding authors.
